# Nutrients-Rich Food Index Scores and the Overall Survival of Ovarian Cancer Patients: Results from the Ovarian Cancer Follow-Up Study, a Prospective Cohort Study

**DOI:** 10.3390/nu15030717

**Published:** 2023-01-31

**Authors:** Jun-Qi Zhao, Qi-Peng Ma, Yi-Fan Wei, Gang Zheng, Bing-Jie Zou, Zong-Da Du, Song Gao, Shi Yan, Xue Qin, Ting-Ting Gong, Yu-Hong Zhao, Qi-Jun Wu

**Affiliations:** 1 Department of Clinical Epidemiology, Shengjing Hospital of China Medical University, Shenyang 110004, China; 2 Clinical Research Center, Shengjing Hospital of China Medical University, Shenyang 110004, China; 3 Key Laboratory of Precision Medical Research on Major Chronic Disease, Shengjing Hospital of China Medical University, Shenyang 110004, China; 4 Department of Obstetrics and Gynecology, Shengjing Hospital of China Medical University, Shenyang 110004, China

**Keywords:** cohort study, nutrient density, nutrients-rich food index scores, ovarian cancer, overall survival

## Abstract

**Background:** The nutrients-rich food (NRF) index provides a score of diet quality. Although high diet quality is associated with survival of ovarian cancer (OC), the associations between NRF index scores and OC survival remain unevaluated. **Methods:** The prospective cohort study enrolled 703 women with newly diagnosed epithelial OC to assess the correlations between NRF index scores and overall survival (OS) in OC patients. Dietary consumption was evaluated through a food frequency questionnaire and diet quality was calculated based on NRF index scores, including three limited nutrients and six (NRF6.3), nine (NRF9.3), or eleven (NRF11.3) benefit nutrients. All-cause deaths were ascertained through medical records combined with active follow-up. Immunohistochemistry (IHC) analyses were conducted to evaluate the expression of IHC indicators (including Estrogen Receptor, Progesterone Receptor, p53, Vimentin, and Wilms’ tumor 1), which were identified by two independent pathologists. The Cox proportional hazards regression models were applied for estimating the hazard ratios (HRs) and 95% confidence intervals (CIs). Moreover, we performed the penalized cubic splines model to assess the curvilinear associations of NRF index scores with OC survival. **Results:** During the median follow-up of 37.17 (interquartile: 24.73–50.17) months, 130 deaths were documented. Compared to the lowest tertiles, the highest tertile of index scores [NRF9.3 (HR = 0.63, 95% CI = 0.41–0.95), NRF6.3 (HR = 0.59, 95% CI = 0.39–0.89), and NRF11.3 (HR = 0.57, 95% CI = 0.38–0.87)] were correlated to better OS, showing an obvious linear trend (all *p* trend < 0.05). Interestingly, the curvilinear association between the NRF6.3 index score and OC survival was also observed (*p* non-linear < 0.05). Subgroup analyses, stratified by clinical, demographic, and IHC features, showed similar risk associations as the unstratified results. Furthermore, there were significant multiplicative interactions between NRF index scores and Progestogen Receptors as well as Wilms’ tumor 1 expressions (all *p* interaction < 0.05). **Conclusions:** Higher NRF index scores were associated with an improved OS in OC patients.

## 1. Introduction

As one of the most deadly gynecological malignancies, the mortality rate of ovarian cancer (OC) ranks first among gynecological malignancies [1]. In 2020, it accounted for an approximated 313,959 new OC cases and 207,252 new deaths worldwide [2]. Given the insidious onset and rapid development of OC [3], the majority of OC patients were diagnosed at the advanced stage [4], and the five-year survival rate of OC patients remained less than 50% [5]. Hence, it is vital to ascertain modifiable factors that could help to improve the prognosis of OC patients. Early evidence suggested that several factors were relevant to the prognosis of OC, such as histological type [6], clinical stage [7], breastfeeding, and menopausal hormone therapy [8]. Nonetheless, these aforementioned factors are difficult to modify. Diet, a potentially modifiable aspect, has an impact on the prognosis of OC, which has been confirmed by numerous epidemiologic studies [9,10,11] and our research [12,13,14]. Clear evidence also indicated that pre-diagnosis high diet quality contributed to improving OC survival [9].

Nutrient density is one of the key elements of diet quality. Nutrients-rich food (NRF) index scores are developed to assess the nutrient density of individual foods, meals, and daily diet based on nutrient composition [15,16]. NRF index scores consist of nutrients-rich (NR) index scores and the limited nutrients (LIM) index score [15]. NR index scores are based on several beneficial nutrients, including macronutrients, Vitamins, and minerals. The LIM index score is based on saturated fatty acids, sodium, and added sugar. NRF index scores are obtained by subtracting the LIM index score from the NR index scores [17]. A previous study suggested a higher NRF9.3 index score was associated with lower all-cause mortality for older people [15]. Some epidemiological studies also indicated that several benefit components of NRF index scores were related to decreased OC risk and better survival [10,18]. For instance, Qin et al. found dietary calcium intake was correlated with a reduced risk of OC [18]. Moreover, a cohort study indicated that pre-diagnosis dietary fiber consumption was correlated to improved OC survival [10]. Additionally, preceding evidence reported that vegetables and fruit, as the main contributors to NRF index scores [15], have been proved to be associated with better OC survival [19]. The aforementioned evidence suggests that high NRF index scores might be associated with better OC survival.

For all we know, no previous literature has investigated the associations of NRF index scores with OC survival. In the present study, we prospectively assess the correlations of NRF index scores with OS of OC patients based on the Ovarian Cancer Follow-Up Study (OOPS).

## 2. Methods

### 2.1. Study Population

**Figure 1:**
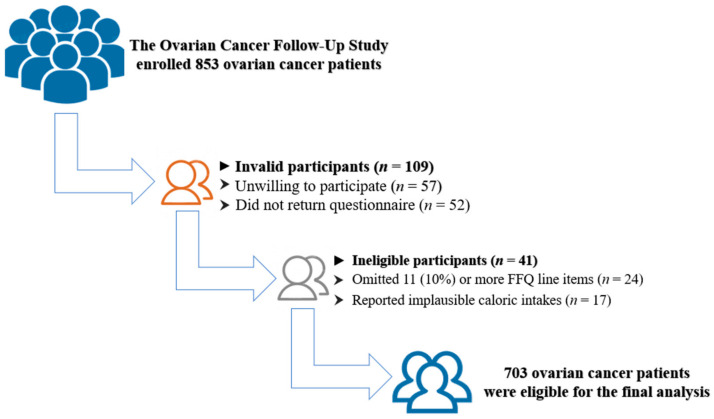
Flow diagram of the selection of participants.

Newly diagnosed OC patients were recruited in the OOPS from January 2015 to December 2020 [20]. Participants meeting the following criteria were included in the present study: (i) epithelial OC confirmed by pathology; (ii) the age of OC patients between 18 and 79 years old; (iii) surgical method was debulking surgery; (iv) enrollment was within 6 months of diagnosis; (v) signed informed consent and volunteered to participate in the study. Briefly, a total of 853 OC women were enrolled. Of these, 57 women refused to continue participation, and 52 women did not return the completed questionnaire. Moreover, we further excluded the women who left out 11 or more FFQ line items (n = 24) or reported unreasonable energy consumption (> 3500 or < 500 kcal/day) (n = 17) from the analysis [14]. Finally, in total, 703 OC patients were available for the present study (Figure 1). Ethical approval was obtained from the Institutional Review Board of the Ethics Committee of Shengjing Hospital of China Medical University, Shenyang, China. All participants signed prior informed consent.

### 2.2. Dietary Exposure Assessment

The dietary intakes of OC patients were collected at recruitment through a validated 111-item food frequency questionnaire (FFQ) with reasonable reliability and validity. For most food items, the reproducibility coefficients (intraclass and spearman) were above 0.5, and the spearman correlation coefficients were 0.3–0.7 between the FFQ and weighed dietary records [21]. All participants were needed to check the usual frequency consumption for each food item with standard serving sizes over 12 months before the diagnosis of OC through the FFQ, which was carried out by skilled and well-trained personnel via face-to-face interviews. Seven response options (i.e., ≥2 times/day; 1 time/day; 4–6 times/week; 2–3 times/week; 1 time/week; 2–3 times/month; and almost never) were provided for participants to choose (Supplementary Table S3). Then, the daily consumption of each food item was estimated by multiplying the frequency consumed per day by the fitted portion size (g/time) [22]. The nutrient consumption was estimated by multiplying the daily intake of each food item by its corresponding nutrient composition according to the Chinese Food Composition Tables (2018) [22,23,24].

NR index scores and the LIM index score were calculated using the sum of the content of NR and LIM in edible portions of 100 kcal foods divided by the daily reference values for NR and LIM according to the 2000-kcal/d diet [25,26], and NRF index scores were calculated through NR index scores by subtracting the LIM index score [17]. Among the different types of NRF index scores, the NRF9.3 index score is the most widely used and extensively tested and validated. Due to the lack of data on dietary Vitamin D, we did not calculate the NR15 and NRF15.3 index scores.

### 2.3. Covariates

Socio-demographic and lifestyle data, including income, education, parity, menopausal status, physical activity (PA), smoking status, alcohol drinking, and dietary change were collected using self-administered questionnaires. Smoking and alcohol drinking status represented smoking or drinking ≥ 1 time/week for more than 6 months. Dietary change represented OC patients who had intentionally changed dietary habits with four response options: this year; 1–2 years ago; 3 years ago; and no. Body weight and height were obtained by well-trained staff with standardized equipment and techniques, subsequently, body mass index (BMI, kg/m^2^) was calculated according to these measurements. All participants were requested to report their duration and usual type of activities in relation to commuting, work, exercise, and housework over the past year [27]. Then, total PA was estimated using metabolic equivalent tasks (METs) of the major PA compendium [28]. The vital clinical data, including residual lesions, age at diagnosis, histological type, International Federation of Gynecology and Obstetrics (FIGO) stage, comorbidities (diabetes, hypertension, coronary heart disease, etc.), and histopathologic grade, were obtained from the electronic medical records of Shengjing hospital.

### 2.4. Immunohistochemistry Analysis

OC and adjacent tissue specimens obtained in the surgery were used for immunohistochemistry (IHC) analysis. First, the specimens firstly formalin-fixed and embedded with a thickness of 3- to 4-μm paraffin. Then, the samples were dewaxed with xylene and hydrated in an ethanol gradient. After that, specimens were quenched using 3% fresh hydrogen peroxide to inhibit peroxidase activity of endogenous tissue, whereafter antigen epitope retrieval was thermally induced with pH 6.0 citrate buffer. Subsequently, these specimens were blocked by normal serum solution and cultivated with primary antibodies against Progesterone Receptor (PR), Estrogen Receptor (ER), p53, Vimentin, and Wilms’ tumor 1 (WT-1) (1:500, Abcam, Cambridge, England) at 4 °C overnight. After washing with PBS, specimens were cultivated with secondary antibodies at 37 °C for 30 min. Ultimately, specimens were disposed of with hematoxylin and diaminobenzidine for coloration and counterstain. IHC indicators were separated into positive and negative expressions according to stained intensity and positively stained cell portion by two independent experienced pathologists.

### 2.5. Follow-Up and Outcome

The interested outcome of the current analysis was overall survival (OS). OC patients were followed up until occurring all-cause death or the end of follow-up (31 March 2021). We ascertained the important features of the participants from medical records and active follow-up. Survival time was calculated as the interval from the histological diagnosis of OC to the end of the follow-up or the date of all-cause death, whichever came first.

### 2.6. Statistical Analysis

The discrepancy in clinical and demographic features by tertiles of NRF9.3 index score was evaluated using the Chi-square test for categorical variables and one-way analysis of variance or the Kruskal–Wallis test for continuous variables. Categorical variables were presented as a number with percentages, whereas continuous variables were shown as means with standard deviation (SD) or medians with interquartile (IQR). The Kaplan–Meier technique was applied to estimate crude survival probabilities and plot crude survival curves. We assessed the proportional hazards assumption by adding interaction terms of each activity variable and the logarithm of survival time, and all variables met the conditions (all *p* > 0.05). In addition, we further verified the proportional hazards assumption with Schoenfeld residuals, the results similarly showed that all variables satisfied the conditions (data not shown). We performed Cox proportional hazards regression models to calculate the hazard ratios (HRs) and corresponding 95% confidence intervals (CIs) for the correlations of NR, LIM, and NRF index scores with OS of OC patients. Continuous index scores were calculated by the increment of per SD. The *p* values for linear trend were calculated by allocating the median value of each tertile for NR, LIM, and NRF index scores as a continuous term in Cox regression models, respectively.

Nutrient density and energy are important ingredients of diet quality, and previous research indicated high diet quality with adequate nutrient intake was correlated to decreased mortality after OC diagnosis [9]. However, evidence about the joint effect of nutrient density and energy on OC survival is limited. Therefore, we explored the joint effect between dietary energy intake and the NRF9.3 index score on OC survival. The cut-off value of dietary energy intake was according to the median of the population. Furthermore, the non-linear correlations between NR, LIM, and NRF index scores and OC survival were tested through the penalized cubic splines model with 3 (i.e., 5, 50, and 95th percentiles) equally spaced knots [29].

More specifically, model 1 was controlled for total energy (continuous, kcal/d) intake and age at diagnosis (<50 or ≥50 years). In model 2, we further controlled for BMI (continuous, kg/m^2^), education (junior college/university or above, senior high school/technical secondary school, and junior secondary or below), monthly household income (<5000, 5000–10,000, ≥10,000 Yuan), menopausal status (yes or no), parity (≤1 or ≥2), alcohol drinking (yes or no), cigarette smoking (yes or no), dietary change (yes or no), and PA (continuous, MET/hours/day) based on model 1. In model 3, we further adjusted for clinical characteristics, including histological type (non-serous or serous), residual lesions (none, <1, and ≥1 cm), FIGO stage (I–II: early FIGO stage; III–IV: advanced FIGO stage; and unknown), histopathologic grade (poorly, moderately, and well differentiated), and comorbidities (yes or no) based on model 2. In addition, we considered including carbohydrates, monounsaturated fatty acids, and polyunsaturated fatty acids in the multivariate-adjusted models. However, due to the multicollinearity between these covariates, they were excluded from the final models.

We similarly conducted multiple stratified analyses to evaluate effect modification by BMI (<25 vs. ≥25 kg/m^2^), age at diagnosis (<50 vs. ≥50 years), menopausal status (“no” vs. “yes”), histological type (serous vs. non-serous), residual lesions (“no” vs. “yes”), FIGO stage (I–II vs. III–IV), PR (“positive” vs. “negative”), ER (“positive” vs. “negative”), p53 expression (“positive” vs. “negative”), Vimentin (“positive” vs. “negative”), and WT-1 (“positive” vs. “negative”). Potential multiplicative interactions between exposure variates and these stratification variates were assessed by introducing cross-product terms in the Cox regression models. Sensitivity analyses were also implemented to verify the stability of our results. Firstly, we excluded the patients with follow-up periods less than one year to assess whether the correlations were independent of the duration of follow-up. Moreover, we excluded the patients with dietary change to alleviate the concern for dietary change on the relationships between NRF index scores and OC survival. We applied SAS software, version 9.4 (SAS Institute, Cary, NC, USA) for all statistical analyses. All tests were two-tailed, and the differences at *p* < 0.05 are considered significant.

## 3. Results

### 3.1. The Components of NR, LIM, and NRF Index Scores

The components of NR, LIM, and NRF index scores are displayed in the Supplementary Table S1. The NR6 index score is based on six nutrients that the USA Food and Drug Administration used to define as healthy foods [16]. The NR9 index score further adds three concerning nutrients (Vitamin E, magnesium, and potassium) recognized by the Dietary Guidelines for Americans, and the NR11 index score adds another five additional nutrients (Vitamin E, Vitamin B_12_, magnesium, potassium, and zinc) of concern for a subset of the population, while the NR15 index score is based on the original Naturally Nutrient Rich score [16,25]. The LIM index score is based on saturated fatty acids, sodium, and added sugar.

### 3.2. Participant Characteristics

Table 1 shows the lifestyle and demographic features of the study population according to tertiles of the NRF9.3 index score. Over the median follow-up period of 37.17 (IQR: 24.73–50.17) months, we ascertained 130 deaths from all causes. The median age at diagnosis of the participants was 53.00 (IQR: 48.00–60.00) years. OC patients with higher NRF9.3 index score had a longer follow-up time and a lower all-cause mortality rate (all *p* < 0.05). Furthermore, patients with higher NRF9.3 index score were prone to postmenopausal syndromes and have more parity (all *p* < 0.05). Moreover, patients with higher NRF9.3 index score tended to consume more whole grains, vegetables, fruit, legumes and legume products, seafood, monounsaturated fatty acids, and polyunsaturated fatty acids, as well as fewer refined grains, desserts, and sugar-containing beverages (all *p* < 0.05). Almost half of OC patients were diagnosed at the advanced FIGO stage (III–IV). Moreover, most OC patients were poorly differentiated (85.21%), serous carcinoma (68.14%), and without residual lesions (78.66%). We noticed that non-serous histological subtypes, larger residual lesions, and advanced FIGO stages were related to poor OC survival (Supplementary Table S2). Meanwhile, negative expressions of ER, PR, and WT-1 were associated with poor OC survival (Supplementary Table S2).

### 3.3. Association between NR, LIM, and NRF Index Score and OC Survival

Table 2 reveals the associations of NR, LIM, and NRF index scores with OS of OC patients. Females with the highest tertile of NRF9.3 index score were correlated to a more favorable survival of OC patients than the lowest tertile (HR _T3 vs. T1_ = 0.63; 95% CI = 0.41–0.95), showing a distinct linear trend (*p* trend < 0.05). Similar patterns were also noticed in the NRF6.3 index score (HR _T3 vs. T1_ = 0.59; 95% CI = 0.39–0.89, *p* trend < 0.05) and the NRF11.3 index score (HR _T3 vs. T1_ = 0.57; 95% CI = 0.38–0.87, *p* trend < 0.05) (Supplementary Figure S1). Moreover, NR6 (HR = 0.63; 95% CI = 0.41–0.96), NR9 (HR = 0.64; 95% CI = 0.42–0.97), and NR11 index scores (HR = 0.63; 95% CI = 0.41–0.96) were similarly related to improved OS of OC patients. However, there were no significant associations of the LIM index score with OS of OC patients in our analyses. Interestingly, a significant curvilinear association of the NRF6.3 index score with OC survival was observed (*p* non-linear < 0.05) (Supplementary Figure S2).

### 3.4. The Joint Effect of NRF Index Score and Dietary Energy Intake on OC Survival

Furthermore, in the joint effect analysis, OC patients with the highest tertile of NRF9.3 index score and lower level of dietary energy intake had favorable OS (HR = 0.45; 95% CI = 0.25–0.83) compared to those with the lowest tertile of NRF9.3 index score and a higher level of dietary energy intake. Moreover, in the subgroup of high energy intake, the OC patients with the highest tertile of NRF9.3 index score had favorable OS (HR = 0.43; 95% CI = 0.24–0.77), compared to the lowest tertile. Similarly, in the lowest tertile of the NRF9.3 index score, the patients with low energy intake were more positively associated with OC survival (HR = 0.52; 95% CI = 0.30–0.92) than the high energy intake (Table 3).

### 3.5. Subgroup Analyses and Sensitivity Analyses

**Figure 2:**
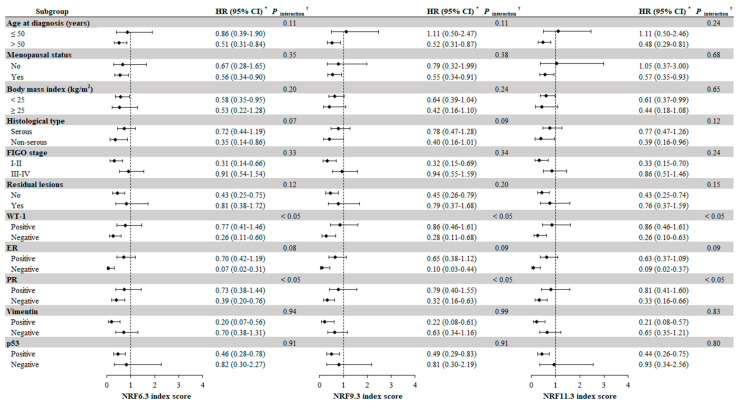
Subgroup analyses of clinical, demographic and immunohistochemical features for the associations of NRF index scores with OS among OC patients. * HRs and 95% CIs were estimated by comparing the highest tertile with the lowest tertile of NRF index scores, using the Cox proportional hazards regression model with adjustment for age at diagnosis, monthly household income, education, parity, menopausal status, alcohol drinking, cigarette smoking, dietary change, BMI, physical activity, histopathologic grade, residual lesions, FIGO stage, histological type, comorbidities, and total energy intake. ^†^ The *p* for interaction was determined by the strata and index scores. Abbreviations: BMI, body mass index; CI, confidence interval; ER, Estrogen Receptor; FIGO, International Federation of Gynecology and Obstetrics; HR, hazard ratio; NRF, nutrients-rich food; OC, ovarian cancer; OS, overall survival; PR, Progestogen Receptor; Ref, reference; WT-1, Wilms’ tumor 1.

The associatons between NRF index scores and OC survival presented in the subgroup of age at diagnosis > 50, early FIGO stage, no residual lesions, or postmenopausal patients were consistent with primary findings. Similar patterns were also present in patients with the positive expressions of p53 and Vimentin and the negative expressions of ER, PR, and WT-1 (Figure 2). Interestingly, we found significant multiplicative interactions of PR and WT-1 expressions with NRF index scores on OC survival (all *p* interaction < 0.05). In sensitivity analyses that excluded the patients with follow-up periods less than one year, we identified the correlations of NRF6.3, NRF9.3, and NRF11.3 index scores with OS of OC patients did not change substantially (Table 4). Similarly, after excluding the patient with dietary change, we found the correlations of NRF6.3, NRF9.3, and NRF11.3 index scores with OC survival remained significant (Table 4).

## 4. Discussion

In this prospective cohort study of 703 women diagnosed with OC, higher NRF index scores were correlated to improved OC survival. Of interest, significant multiplicative interactions were observed between the expressions of PR as well as WT-1 and NRF index scores on OC survival. More importantly, the combination of a higher NRF9.3 index score with lower dietary energy intake was correlated to improved survival of OC patients.

As far as we know, no previous research has examined the correlations of NRF index scores with the survival of OC patients. Recently, only one publication has investigated the relationships of the NRF9.3 index score with cardiovascular disease (CVD) incidence and all-cause mortality [15]. Streppel et al. performed a cohort study with 4969 older persons, and found the NRF9.3 index score was related to a decreased all-cause mortality (HR _Q4 vs Q1_ = 0.84, 95% CI = 0.74–0.96), while a null significant association was found between CVD risk and NRF9.3 index score [15]. Epidemiological studies about NRF index scores and health outcomes are still limited. Nonetheless, a large body of studies has presented indirect evidence for NRF index scores and OC survival. In the present study, vegetables and fruit were the major food items responsible for the NRF9.3 index score. Previous studies indicated that these food items were associated with better OC survival [10,19,30]. For example, a cohort study included 811 invasive OC women suggested that higher green leafy vegetables were related to favorable OC survival (HR = 0.79; 95% CI = 0.62–0.99) [10]. Moreover, Wei et al. observed that higher pre-diagnosis intake of cruciferous vegetables were positively correlated to OS of OC patients (HR = 0.57; 95% CI = 0.33–0.98) [30]. Additionally, a meta-analysis indicated pre-diagnosis fruit consumption was correlated to the decreased all-cause mortality of patients with OC (HR = 0.82; 95% CI = 0.70–0.96) [19]. These findings provided indirect evidence for NRF index scores and OC survival. In general, relevant research is still limited, further investigation is warranted to prove the relationships between NRF index scores and OC survival.

More importantly, we noticed that a higher NRF9.3 index score and a lower level of dietary energy intake were correlated to improved survival of OC patients. NRF index scores as indices reflected nutrient density, although no previous study investigated the joint effect of NRF9.3 index score and dietary energy intake on OC survival, several studies provided evidence for the joint effect of nutrient density and energy intake on the survival of cancer. Previous literature indicated that the Mediterranean diet was correlated to lower dietary energy intake and higher nutrient density [31,32], which has been proved to be significantly related to lower all-cause cancer mortality risk [33]. Moreover, the vitro experiments suggested that high energy intake promoted OC progression and provided energy for rapid tumor growth [34], while adequate nutrient intake could inhibit OC cell proliferation and contribute to improved OC survival [35,36,37,38,39]. Therefore, lower dietary energy and higher nutrient density intake might be correlated to better OC survival. Given the lack of relevant research, further research is warranted to verify our results.

Existing evidence suggested that IHC biomarker expressions, including PR, ER, p53, and Vimentin, might exert a prognostic impact on the survival of females with OC [40,41], while WT-1 is a highly specific and sensitive IHC biomarker for diagnosing ovarian high-grade serous carcinomas [42]. For instance, Sieh et al. have shown that the positive expression of PR was correlated to improved survival of OC [40]. Of note, we found NRF index scores were associated with better OC survival in the subgroup of the negative expression of PR. Moreover, significant multiplicative interactions were also noticed between NRF index scores and PR expression on the survival of females with OC. The above evidence hinted that dietary NRF index scores might interact with PR expression on the survival of OC patients. In addition, dietary NRF index scores might alleviate the adverse effect of the negative expression of PR with OC survival, in that lower HRs were found among patients with higher dietary NRF index scores. However, restricted to the small sample size of some categories, the possibility of accidental findings could not be completely eliminated. Further research with a large study population is needed for confirmation.

The exact biological mechanisms underlying NRF Index scores and OC survival have not been fully established. A potential explanation for our results is that several benefit components of NRF index scores might be related to improved OC survival. Vitro experiments showed that a high level of calcium intake might decrease OC risk by downgrading the circulating parathyroid hormone (PTH) [43,44,45]. Meanwhile, the down-regulation of PTH could decrease osteoblastic and hepatic insulin-like growth factor-1 (IGF-1) synthesis, which could subsequently promote apoptosis, decrease proliferation, and attenuated the mitogenesis of OC cells [39,44,46,47]. Moreover, iron reduced OC cell survival with Ras/MAPK dependent way and via promoting mitochondrial damage [48,49], and magnesium could inhibit cancer cell growth through regulation of cell proliferation, differentiation, apoptosis, maintaining genomic stability, and prevention of angiogenesis [38]. Additionally, Vitamin B_12_ has an impact on DNA synthesis, methylation, and redox metabolism, which might influence the pathways enhancing OC cell proliferation [35]. Furthermore, antioxidant Vitamins, including Vitamins A, C, and E, might improve OC survival by alleviating DNA damage, suppressing cell proliferation, regulating cell apoptosis and differentiation, and increasing immune function [36,37,50]. In addition, the combination of Vitamin C with paclitaxel and carboplatin also synergistically inhibited OC in mouse models and relieved chemotherapy-related toxicity of OC patients [37]. On the contrary, the limited nutrients exerted an adverse effect on OC survival. High-added sugars induced the synthesis of IGF-I and insulin might promote the development of tumors by stimulating the synthesis of sex steroids, inhibiting apoptosis, and promoting cell proliferation [51] or by facilitating the generation of vascular endothelial growth factor, which promotes tumor cell migration and supports tumor growth [52]. Future research should further explore and illustrate the detailed and exact biological mechanisms of the combination of these benefit components and limited nutrients on OC survival.

Our investigation has several strengths. The present study has satisfactory innovativeness and provides the first report about the relationships between NRF index scores and OS of OC patients. Additionally, the strengths of our study, including prospective design, high participation rates (over 90%) and follow-up retention rates (over 90%), result in the decreased possibility of selection bias and recall bias. Furthermore, we collected detailed comprehensive lifestyle and clinical features with regard to OC survival and rigorously adjusted these potential confounding factors in the present study, which provides more credible results. We also performed multiple subgroup analyses and sensitivity analyses, and the results are consistent with the primary findings, which further enhance the reliability of our study.

Meanwhile, several limitations should be mentioned when interpreting our findings. First, data on diet was obtained by the FFQ, which could result in the misestimate of various nutrient intake. However, the FFQ was validated by our previous studies with reasonable validity and reliability and conducted by skilled and well-trained personnel via face-to-face interviews, which could alleviate the concern. Second, the present study only collected dietary information about one year before diagnosis, while some OC patients might change their diet habits before diagnosis. Nonetheless, only 23.9% of OC patients reported they had changed dietary habits, and dietary change was adjusted in the multivariate-adjusted model. Additionally, we conducted sensitivity analyses that excluded the patients with dietary change, and the results remained significant. Third, as the information on the reason for the death of OC patients was not available, we did not examine the correlations of NRF index scores with OC-specific mortality. However, previous literature suggested that the results of all-cause mortality were highly consistent with OC-specific mortality [9]. Fourth, although the information about chemotherapy and surgery had been collected, the detailed information is relatively limited, and residual confounders from diverse chemotherapy and surgical regimens on the relationships of NRF index scores with the survival OC patients could not have been completely removed. Fifth, as a single-center cohort study and all participants are Chinese, it should be cautiously interpreted when generalizing our results to other populations. Last, although we adjusted for many potentially confounding variables, the influence of unmeasured or residual confounders could not be removed in any observational studies.

## 5. Conclusions

Collectively, findings from the present cohort study underscore that NRF index scores are positively correlated to better survival of OC patients. Meanwhile, we notice the OC patients with the highest NRF9.3 index score and lower dietary energy intake have better survival. Further research is needed to confirm the current findings.

## Figures and Tables

**Table 1:** Baseline characteristics of females with ovarian cancer by tertiles of NRF9.3 index score (N = 703).

Characteristics	Tertiles of NRF9.3 Index Score			*p* Value *
	T1	T2	T3	
Range	<36.48	36.48–≤46.39	≥46.39	
No. of deaths/patients	56/234	31/234	43/235	<0.05
Median (IQR) Age at diagnosis (years)	53.00 (46.00–61.00)	53.00 (48.00–59.00)	54.00 (48.00–61.00)	0.39
Median (IQR) Follow-up time (months)	28.35 (17.80–42.17)	31.67 (21.43–45.50)	34.37 (22.50–49.90)	<0.05
Median (IQR) Body mass index (kg/m^2^)	23.30 (21.00–25.20)	23.30 (21.00–25.10)	22.90 (20.40–24.80)	0.17
Median (IQR) Physical activity (MET/hours/day)	14.30 (7.00–22.70)	14.65 (6.20–21.50)	13.20 (6.20–22.40)	0.64
Ever cigarette smoking	26 (11.11)	16 (6.84)	26 (11.06)	0.20
Ever alcohol drinking	56 (23.93)	50 (21.37)	43 (18.30)	0.33
Ever dietary change	50 (21.37)	57 (24.36)	61 (25.96)	0.49
Ever menopause	163 (69.66)	160 (68.38)	185 (78.72)	<0.05
Parity				<0.05
≤1	184 (78.63)	152 (64.96)	169 (71.91)	
≥2	50 (21.37)	82 (35.04)	66 (28.09)	
Educational level				0.22
Junior secondary or below	121 (51.71)	117 (50.00)	137 (58.30)	
Senior high school/technical secondary school	53 (22.65)	56 (23.93)	38 (16.17)	
Junior college/university or above	60 (25.64)	61 (26.07)	60 (25.53)	
Income per month (Yuan)				0.46
<5000	147 (62.82)	131 (55.98)	143 (60.85)	
5000 to 10,000	63 (26.92)	67 (28.64)	64 (27.23)	
≥10,000	24 (10.26)	36 (15.38)	28 (11.92)	
Mean (SD) total energy (kcal/d)	1413.99 (547.89)	1448.03 (582.17)	1505.03 (525.01)	0.20
Mean (SD) refined grains (g/d)	612.58 (214.63)	604.44 (215.94)	523.58 (216.06)	<0.05
Mean (SD) whole grains (g/d)	13.20 (18.97)	17.07 (21.50)	19.67 (19.91)	<0.05
Mean (SD) vegetables (g/d)	126.74 (65.55)	187.42 (82.53)	284.98 (115.68)	<0.05
Mean (SD) fruit (g/d)	114.10 (91.25)	176.62 (141.24)	256.38 (178.31)	<0.05
Mean (SD) legumes and legume products (g/d)	38.12 (41.23)	63.50 (59.31)	107.41 (91.59)	<0.05
Mean (SD) meat (g/d)	37.61 (29.61)	41.03 (35.31)	38.25 (31.12)	0.47
Mean (SD) seafood (g/d)	18.70 (20.10)	28.58 (29.93)	38.24 (35.63)	<0.05
Mean (SD) desserts (g/d)	27.90 (40.24)	19.15 (30.17)	12.67 (19.25)	<0.05
Mean (SD) sugar-containing beverages (g/d)	54.00 (120.70)	24.89 (64.81)	19.90 (51.80)	<0.05
Mean (SD) carbohydrates (g/d)	226.24 (75.21)	226.74 (81.49)	227.71 (78.63)	0.98
Mean (SD) monounsaturated fatty acids (g/d)	8.62 (5.43)	9.26 (6.02)	9.96 (5.48)	<0.05
Mean (SD) polyunsaturated fatty acids (g/d)	4.01 (2.26)	5.18 (3.03)	6.69 (3.60)	<0.05

Abbreviations: IQR, interquartile; MET, metabolic equivalents of task; NRF, nutrients-rich food; SD, standard deviation; T, tertile. * *p* values were determined using a Chi-square test for categorical variables, and one-way ANOVA or Kruskal–Wallis for continuous variables. Values are numbers (percentages) unless stated otherwise.

**Table 2:** The associations of NR, LIM, and NRF index scores with overall survival of 703 ovarian cancer patients *.

Characteristics		Deaths, N (% of Total Deaths)	Multivariable-Adjusted Models		
			Model 1 ^a^	Model 2 ^b^	Model 3 ^c^
**NRF6.3** **index score**	T1 (<25.27)	59 (25.21)	1.00 (Ref)	1.00 (Ref)	1.00 (Ref)
	T2 (25.27–≤32.48)	28 (11.97)	0.43 (0.27–0.67)	0.40 (0.25–0.64)	0.38 (0.23–0.61)
	T3 (≥32.48)	43 (18.30)	0.65 (0.44–0.96)	0.60 (0.40–0.90)	0.59 (0.39–0.89)
	Continuous **		0.82 (0.68–0.98)	0.80 (0.67–0.97)	0.79 (0.65–0.96)
	*p* for trend ^†^		<0.05	<0.05	<0.05
**NRF9.3** **index score**	T1 (<36.48)	56 (23.93)	1.00 (Ref)	1.00 (Ref)	1.00 (Ref)
	T2 (36.48–≤46.39)	31 (13.25)	0.51 (0.33–0.79)	0.49 (0.31–0.76)	0.44 (0.28–0.70)
	T3 (≥46.39)	43 (18.30)	0.68 (0.45–1.01)	0.63 (0.42–0.94)	0.63 (0.41–0.95)
	Continuous **		0.83 (0.69–0.99)	0.82 (0.68–0.99)	0.81 (0.67–0.98)
	*p* for trend ^†^		0.07	<0.05	<0.05
**NRF11.3** **index score**	T1 (<40.75)	58 (24.79)	1.00 (Ref)	1.00 (Ref)	1.00 (Ref)
	T2 (40.75–≤50.78)	31 (13.25)	0.49 (0.32–0.76)	0.47 (0.30–0.73)	0.44 (0.28–0.69)
	T3 (≥50.78)	41 (17.45)	0.61 (0.41–0.91)	0.57 (0.38–0.86)	0.57 (0.38–0.87)
	Continuous **		0.83 (0.69–0.99)	0.82 (0.68–0.99)	0.81 (0.67–0.98)
	*p* for trend ^†^		<0.05	<0.05	<0.05
**NR6** **index score**	T1 (<32.38)	55 (23.50)	1.00 (Ref)	1.00 (Ref)	1.00 (Ref)
	T2 (32.38–≤39.76)	34 (14.53)	0.53 (0.34–0.82)	0.55 (0.35–0.85)	0.52 (0.33–0.82)
	T3 (≥39.76)	41 (17.45)	0.65 (0.43–0.98)	0.61 (0.40–0.93)	0.63 (0.41–0.96)
	Continuous **		0.85 (0.71–1.01)	0.84 (0.69–1.01)	0.82 (0.67–0.99)
	*p* for trend ^†^		<0.05	<0.05	<0.05
**NR9** **index score**	T1 (<43.75)	54 (23.08)	1.00 (Ref)	1.00 (Ref)	1.00 (Ref)
	T2 (43.75–≤53.85)	34 (14.53)	0.54 (0.35–0.84)	0.54 (0.35–0.85)	0.54 (0.34–0.85)
	T3 (≥53.85)	42 (32.31)	0.66 (0.44–0.99)	0.63 (0.41–0.96)	0.64 (0.42–0.97)
	Continuous **		0.85 (0.71–1.02)	0.85 (0.71–1.02)	0.83 (0.69–1.01)
	*p* for trend ^†^		0.07	<0.05	0.06
**NR11** **index score**	T1 (<48.12)	55 (23.50)	1.00 (Ref)	1.00 (Ref)	1.00 (Ref)
	T2 (48.12–≤58.24)	33 (14.10)	0.53 (0.34–0.82)	0.52 (0.33–0.82)	0.52 (0.33–0.81)
	T3 (≥58.24)	42 (17.87)	0.65 (0.43–0.98)	0.62 (0.41–0.94)	0.63 (0.41–0.96)
	Continuous **		0.85 (0.71–1.02)	0.85 (0.71–1.02)	0.83 (0.69–1.01)
	*p* for trend ^†^		0.06	<0.05	0.05
**LIM** **index score**	T1 (<6.05)	36 (15.38)	1.00 (Ref)	1.00 (Ref)	1.00 (Ref)
	T2 (6.05–≤8.27)	43 (18.38)	1.10 (0.70–1.72)	1.05 (0.67–1.65)	1.02 (0.65–1.61)
	T3 (≥8.27)	51 (21.70)	1.48 (0.94–2.32)	1.52 (0.97–2.40)	1.42 (0.89–2.26)
	Continuous **		1.12 (0.97–1.40)	1.18 (0.98–1.43)	1.19 (0.98–1.44)
	*p* for trend ^†^		0.08	0.06	0.12

Abbreviations: BMI, body mass index; CI, confidence interval; FIGO, International Federation of Gynecology and Obstetrics; HR, hazard ratio; LIM, limited nutrients; NR, nutrients-rich; NRF, nutrients-rich food; Ref, reference; T, tertile. * HRs and 95% CIs were estimated using the Cox proportional hazards regression model. ** Continuous NR, LIM, and NRF index scores were estimated by per SD increment. ^†^ Test for trends based on variables containing the median value for each tertile. ^a^ Adjusted for total energy intake (continuous, kcal/day) and age at diagnosis (<50 or ≥50 years). ^b^ Based on model 1 and further adjusted for monthly household income (<5000, 5000–10,000, ≥10,000 CNY), education (junior secondary or below, senior high school/technical secondary school, and junior college/university or above), parity (≤1 or ≥2), menopausal status (yes or no), alcohol drinking (yes or no), cigarette smoking (yes or no), dietary change (yes or no), BMI (continuous, kg/m^2^), and physical activity (continuous, MET/hours/day). ^c^ Based on model 2 and further adjusted for histopathologic grade (well, moderately, and poorly differentiated), residual lesions (none, <1, and ≥1 cm), FIGO stage (I–II, III–IV, and unknown), histological type (serous or non-serous), and comorbidities (yes or no).

**Table 3:** The joint effect between dietary energy intake and NRF9.3 index score on the overall survival of ovarian cancer patients *.

Variables		Dietary Energy Intake (kcal/d) ^†^	
		High	Low
**NRF9.3 index score**	T1 (<36.48)	1.00 (Ref)	0.52 (0.30–0.92)
	T2 (36.48–≤46.39)	0.36 (0.19–0.68)	0.27 (0.14–0.53)
	T3 (≥46.39)	0.43 (0.24–0.77)	0.45 (0.25–0.83)

Abbreviations: BMI, body mass index; CI, confidence interval; FIGO, International Federation of Gynecology and Obstetrics; HR, hazard ratio; Ref, reference; NRF, nutrients-rich food; T, tertile. * HRs and 95% CIs were estimated with the use of the Cox proportional hazards regression model with adjustment for age at diagnosis, monthly household income, education, parity, menopausal status, alcohol drinking, cigarette smoking, dietary change, BMI, physical activity, histopathologic grade, residual lesions, FIGO stage, histological type, and comorbidities. ^†^ High and low dietary energy intake were divided according to the median dietary energy intake (1370.73 kcal/d).

**Table 4:** Sensitivity analyses: the associations of tertiles of NRF index scores with overall survival of ovarian cancer patients.

Characteristics	Excluding Deaths Occurring in One Year of Follow-Up *		Excluding Patients with Dietary Change **	
	Range	HR (95% CI)	Range	HR (95% CI)
**NRF6.3 index score**	T1 (<25.30)	1.00 (Ref)	T1 (<25.22)	1.00 (Ref)
	T2 (25.30–≤32.48)	0.43 (0.25–0.75)	T2 (25.22–≤32.39)	0.43 (0.25–0.73)
	T3 (≥32.48)	0.55 (0.34–0.91)	T3 (≥32.39)	0.53 (0.33–0.87)
	*p* trend ^†^	<0.05	*p* trend ^†^	<0.05
**NRF9.3 index score**	T1 (<36.52)	1.00 (Ref)	T1 (<36.24)	1.00 (Ref)
	T2 (36.52–≤46.38)	0.44 (0.26–0.75)	T2 (36.24–≤46.25)	0.44 (0.26–0.75)
	T3 (≥46.38)	0.59 (0.35–0.97)	T3 (≥46.25)	0.55 (0.34–0.89)
	*p* trend ^†^	0.05	*p* trend ^†^	<0.05
**NRF11.3 index score**	T1 (<40.89)	1.00 (Ref)	T1 (<40.52)	1.00 (Ref)
	T2 (40.89–≤50.78)	0.52 (0.31–0.90)	T2 (40.52–≤50.61)	0.49 (0.29–0.82)
	T3 (≥50.78)	0.52 (0.31–0.88)	T3 (≥50.61)	0.59 (0.36–0.96)
	*p* trend ^†^	<0.05	*p* trend ^†^	<0.05

Abbreviations: BMI, body mass index; CI, confidence interval; FIGO, International Federation of Gynecology and Obstetrics; HR, hazard ratio; NRF, nutrients-rich food; Ref, reference; T, tertile. * HRs and 95% Cis were estimated with the use of the Cox proportional hazards regression model with adjustment for age at diagnosis, monthly household income, education, parity, menopausal status, alcohol drinking, cigarette smoking, dietary change, BMI, physical activity, histopathologic grade, residual lesions, FIGO stage, histological type, comorbidities, and total energy intake. ** HRs and 95% Cis were estimated with the use of the Cox proportional hazards regression model with adjustment for age at diagnosis, monthly household income, education, parity, menopausal status, alcohol drinking, cigarette smoking, BMI, physical activity, histopathologic grade, residual lesions, FIGO stage, histological type, comorbidities, and total energy intake. ^†^ The *p* for trend was determined by variables containing the median value for each tertile.

## Data Availability

The original contributions presented in the study are included in the article/supplementary material, further inquiries can be directed to the corresponding authors.

## Supplementary Materials

The following supporting information can be downloaded at: https://www.mdpi.com/article/10.3390/nu15030717/s1. Supplementary Table S1. The components of NR, LIM, and NRF index scores; Supplementary Table S2. Selected clinical and immunohistochemical characteristics and associations with overall survival among ovarian cancer patients; Supplementary Table S3. Data collection on the consumption of vegetables and fruit; Supplementary Figure S1. Kaplan–Meier survival curves for the NRF6.3 index score (A), the NRF9.3 index score (B), the NRF11.3 index score (C); Supplementary Figure S2. HRs and 95% CIs of overall survival among OC patients by NRF6.3 index score (A), NRF9.3 index score (B), and NRF11.3 index score (C).

## References

[1] Menon U., Karpinskyj C., Gentry-Maharaj A. (2018). Ovarian Cancer Prevention and Screening. Obstet. Gynecol..

[2] Sung H., Ferlay J., Siegel R.L., Laversanne M., Soerjomataram I., Jemal A., Bray F. (2021). Global Cancer Statistics 2020: GLOBOCAN Estimates of Incidence and Mortality Worldwide for 36 Cancers in 185 Countries. CA Cancer J. Clin..

[3] Torre L.A., Trabert B., DeSantis C.E., Miller K.D., Samimi G., Runowicz C.D., Gaudet M.M., Jemal A., Siegel R.L. (2018). Ovarian cancer statistics, 2018. CA Cancer J. Clin..

[4] Doubeni C.A., Doubeni A.R., Myers A.E. (2016). Diagnosis and Management of Ovarian Cancer. Am. Fam. Physician.

[5] Moufarrij S., Dandapani M., Arthofer E., Gomez S., Srivastava A., Lopez-Acevedo M., Villagra A., Chiappinelli K.B. (2019). Epigenetic therapy for ovarian cancer: Promise and progress. Clin. Epigenetics.

[6] Peres L.C., Cushing-Haugen K.L., Köbel M., Harris H.R., Berchuck A., Rossing M.A., Schildkraut J.M., Doherty J.A. (2019). Invasive Epithelial Ovarian Cancer Survival by Histotype and Disease Stage. J. Natl. Cancer Inst..

[7] Chudecka-Głaz A., Cymbaluk-Płoska A., Luterek-Puszyńska K., Menkiszak J. (2016). Diagnostic usefulness of the Risk of Ovarian Malignancy Algorithm using the electrochemiluminescence immunoassay for HE4 and the chemiluminescence microparticle immunoassay for CA125. Oncol. Lett..

[8] Bešević J., Gunter M.J., Fortner R.T., Tsilidis K.K., Weiderpass E., Onland-Moret N.C., Dossus L., Tjønneland A., Hansen L., Overvad K. (2015). Reproductive factors and epithelial ovarian cancer survival in the EPIC cohort study. Br. J. Cancer.

[9] Thomson C.A., Crane T.E., Wertheim B.C., Neuhouser M.L., Li W., Snetselaar L.G., Basen-Engquist K.M., Zhou Y., Irwin M.L. (2014). Diet quality and survival after ovarian cancer: Results from the Women’s Health Initiative. J. Natl. Cancer Inst..

[10] Playdon M.C., Nagle C.M., Ibiebele T.I., Ferrucci L.M., Protani M.M., Carter J., Hyde S.E., Neesham D., Nicklin J.L., Mayne S.T. (2017). Pre-diagnosis diet and survival after a diagnosis of ovarian cancer. Br. J. Cancer.

[11] Sasamoto N., Wang T., Townsend M.K., Eliassen A.H., Tabung F.K., Giovannucci E.L., Matulonis U.A., Terry K.L., Tworoger S.S., Harris H.R. (2022). Pre-diagnosis and post-diagnosis dietary patterns and survival in women with ovarian cancer. Br. J. Cancer.

[12] Wen Z.Y., Liu C., Liu F.H., Wei Y.F., Xu H.L., Wang R., Li X.Y., Li Y.Z., Yan S., Qin X. (2022). Association between pre-diagnostic dietary pattern and survival of ovarian cancer: Evidence from a prospective cohort study. Clin. Nutr..

[13] Wei Y.F., Sun M.L., Wen Z.Y., Liu F.H., Liu Y.S., Yan S., Qin X., Gao S., Li X.Q., Zhao Y.H. (2022). Pre-diagnosis meat intake and cooking method and ovarian cancer survival: Results from the Ovarian Cancer Follow-Up Study (OOPS). Food Funct..

[14] Zhao J.Q., Hao Y.Y., Gong T.T., Wei Y.F., Zheng G., Du Z.D., Zou B.J., Yan S., Liu F.H., Gao S. (2022). Phytosterol intake and overall survival in newly diagnosed ovarian cancer patients: An ambispective cohort study. Front. Nutr..

[15] Streppel M.T., Sluik D., van Yperen J.F., Geelen A., Hofman A., Franco O.H., Witteman J.C., Feskens E.J. (2014). Nutrient-rich foods, cardiovascular diseases and all-cause mortality: The Rotterdam study. Eur. J. Clin. Nutr..

[16] Drewnowski A. (2009). Defining nutrient density: Development and validation of the nutrient rich foods index. J. Am. Coll. Nutr..

[17] Fulgoni V.L., Keast D.R., Drewnowski A. (2009). Development and validation of the nutrient-rich foods index: A tool to measure nutritional quality of foods. J. Nutr..

[18] Qin B., Moorman P.G., Alberg A.J., Barnholtz-Sloan J.S., Bondy M., Cote M.L., Funkhouser E., Peters E.S., Schwartz A.G., Terry P. (2016). Dairy, calcium, vitamin D and ovarian cancer risk in African-American women. Br. J. Cancer.

[19] Hurtado-Barroso S., Trius-Soler M., Lamuela-Raventós R.M., Zamora-Ros R. (2020). Vegetable and Fruit Consumption and Prognosis Among Cancer Survivors: A Systematic Review and Meta-Analysis of Cohort Studies. Adv. Nutr..

[20] Gong T.T., Liu F.H., Liu Y.S., Yan S., Xu H.L., He X.H., Wei Y.F., Qin X., Gao S., Zhao Y.H. (2022). A Follow-Up Study of Ovarian Cancer (OOPS): A Study Protocol. Front. Nutr..

[21] Cui Q., Xia Y., Liu Y., Sun Y., Ye K., Li W., Wu Q., Chang Q., Zhao Y. (2022). Validity and reproducibility of a FFQ for assessing dietary intake among residents of northeast China: Northeast cohort study of China. Br. J. Nutr..

[22] Hehua Z., Yang X., Qing C., Shanyan G., Yuhong Z. (2021). Dietary patterns and associations between air pollution and gestational diabetes mellitus. Environ. Int..

[23] Yang Y.X., Wang Y.G., He M., Pan X.C., Wang Z. (2018). China Food Composition (Standard Edition).

[24] Hu Y., Ding M., Yuan C., Wu K., Smith-Warner S.A., Hu F.B., Chan A.T., Meyerhardt J.A., Ogino S., Fuchs C.S. (2018). Association Between Coffee Intake after Diagnosis of Colorectal Cancer and Reduced Mortality. Gastroenterology.

[25] Drewnowski A. (2005). Concept of a nutritious food: Toward a nutrient density score. Am. J. Clin. Nutr..

[26] Maillot M., Darmon N., Darmon M., Lafay L., Drewnowski A. (2007). Nutrient-dense food groups have high energy costs: An econometric approach to nutrient profiling. J. Nutr..

[27] Du H., Bennett D., Li L., Whitlock G., Guo Y., Collins R., Chen J., Bian Z., Hong L.S., Feng S. (2013). Physical activity and sedentary leisure time and their associations with BMI, waist circumference, and percentage body fat in 0.5 million adults: The China Kadoorie Biobank study. Am. J. Clin. Nutr..

[28] Ainsworth B.E., Haskell W.L., Herrmann S.D., Meckes N., Bassett D.R., Tudor-Locke C., Greer J.L., Vezina J., Whitt-Glover M.C., Leon A.S. (2011). 2011 Compendium of Physical Activities: A second update of codes and MET values. Med. Sci. Sport. Exerc..

[29] Desquilbet L., Mariotti F. (2010). Dose-response analyses using restricted cubic spline functions in public health research. Stat. Med..

[30] Wei Y.F., Hao Y.Y., Gao S., Li X.Q., Liu F.H., Wen Z.Y., Wang H.Y., Zhang S., Yan S., Luan M. (2021). Pre-diagnosis Cruciferous Vegetables and Isothiocyanates Intake and Ovarian Cancer Survival: A Prospective Cohort Study. Front. Nutr..

[31] Peng W., Berry E.M., Goldsmith R. (2019). Adherence to the Mediterranean diet was positively associated with micronutrient adequacy and negatively associated with dietary energy density among adolescents. J. Hum. Nutr. Diet. Off. J. Br. Diet. Assoc..

[32] Cano-Ibáñez N., Gea A., Ruiz-Canela M., Corella D., Salas-Salvadó J., Schröder H., Navarrete-Muñoz E.M., Romaguera D., Martínez J.A., Barón-López F.J. (2020). Diet quality and nutrient density in subjects with metabolic syndrome: Influence of socioeconomic status and lifestyle factors. A cross-sectional assessment in the PREDIMED-Plus study. Clin. Nutr..

[33] Schwingshackl L., Hoffmann G. (2015). Adherence to Mediterranean diet and risk of cancer: An updated systematic review and meta-analysis of observational studies. Cancer Med..

[34] Nieman K.M., Kenny H.A., Penicka C.V., Ladanyi A., Buell-Gutbrod R., Zillhardt M.R., Romero I.L., Carey M.S., Mills G.B., Hotamisligil G.S. (2011). Adipocytes promote ovarian cancer metastasis and provide energy for rapid tumor growth. Nat. Med..

[35] Oliai Araghi S., Kiefte-de Jong J.C., van Dijk S.C., Swart K.M.A., van Laarhoven H.W., van Schoor N.M., de Groot L., Lemmens V., Stricker B.H., Uitterlinden A.G. (2019). Folic Acid and Vitamin B12 Supplementation and the Risk of Cancer: Long-term Follow-up of the B Vitamins for the Prevention of Osteoporotic Fractures (B-PROOF) Trial. Cancer Epidemiol Biomarkers Prev..

[36] Tanaka T., Shnimizu M., Moriwaki H. (2012). Cancer chemoprevention by carotenoids. Molecules.

[37] Ma Y., Chapman J., Levine M., Polireddy K., Drisko J., Chen Q. (2014). High-dose parenteral ascorbate enhanced chemosensitivity of ovarian cancer and reduced toxicity of chemotherapy. Sci. Transl. Med..

[38] Costello R.B., Rosanoff A., Dai Q., Saldanha L.G., Potischman N.A. (2021). Perspective: Characterization of Dietary Supplements Containing Calcium and Magnesium and Their Respective Ratio-Is a Rising Ratio a Cause for Concern?. Adv. Nutr..

[39] Renehan A.G., Zwahlen M., Minder C., O’Dwyer S.T., Shalet S.M., Egger M. (2004). Insulin-like growth factor (IGF)-I, IGF binding protein-3, and cancer risk: Systematic review and meta-regression analysis. Lancet.

[40] Sieh W., Köbel M., Longacre T.A., Bowtell D.D., deFazio A., Goodman M.T., Høgdall E., Deen S., Wentzensen N., Moysich K.B. (2013). Hormone-receptor expression and ovarian cancer survival: An Ovarian Tumor Tissue Analysis consortium study. Lancet Oncol..

[41] Dionísio de Sousa I.J., Marques D.S., Príncipe C., Portugal R.V., Canberk S., Prazeres H., Lopes J.M., Gimba E.R.P., Lima R.T., Soares P. (2020). Predictive Biomarkers and Patient Outcome in Platinum-Resistant (PLD-Treated) Ovarian Cancer. Diagnostics.

[42] Rekhi B., Deodhar K.K., Menon S., Maheshwari A., Bajpai J., Ghosh J., Shylasree S.T., Gupta S. (2018). Napsin A and WT 1 are useful immunohistochemical markers for differentiating clear cell carcinoma ovary from high-grade serous carcinoma. APMIS Acta Pathol. Microbiol. Immunol. Scand..

[43] Goodman M.T., Wu A.H., Tung K.H., McDuffie K., Cramer D.W., Wilkens L.R., Terada K., Reichardt J.K., Ng W.G. (2002). Association of galactose-1-phosphate uridyltransferase activity and N314D genotype with the risk of ovarian cancer. Am. J. Epidemiol..

[44] McCarty M.F. (2000). Parathyroid hormone may be a cancer promoter—An explanation for the decrease in cancer risk associated with ultraviolet light, calcium, and vitamin D. Med. Hypotheses.

[45] Ramasamy I. (2006). Recent advances in physiological calcium homeostasis. Clin. Chem. Lab. Med..

[46] Khandwala H.M., McCutcheon I.E., Flyvbjerg A., Friend K.E. (2000). The effects of insulin-like growth factors on tumorigenesis and neoplastic growth. Endocr. Rev..

[47] Lukanova A., Lundin E., Toniolo P., Micheli A., Akhmedkhanov A., Rinaldi S., Muti P., Lenner P., Biessy C., Krogh V. (2002). Circulating levels of insulin-like growth factor-I and risk of ovarian cancer. Int. J. Cancer.

[48] Bauckman K.A., Haller E., Flores I., Nanjundan M. (2013). Iron modulates cell survival in a Ras- and MAPK-dependent manner in ovarian cells. Cell Death Dis..

[49] Bauckman K., Haller E., Taran N., Rockfield S., Ruiz-Rivera A., Nanjundan M. (2015). Iron alters cell survival in a mitochondria-dependent pathway in ovarian cancer cells. Biochem. J..

[50] Cramer D.W., Kuper H., Harlow B.L., Titus-Ernstoff L. (2001). Carotenoids, antioxidants and ovarian cancer risk in pre- and postmenopausal women. Int. J. Cancer.

[51] Kaaks R., Lukanova A. (2001). Energy balance and cancer: The role of insulin and insulin-like growth factor-I. Proc. Nutr. Soc..

[52] Bustin S.A., Jenkins P.J. (2001). The growth hormone-insulin-like growth factor-I axis and colorectal cancer. Trends Mol. Med..

