# Supplementary material for: Nutrients-Rich Food Index Scores and the Overall Survival of Ovarian Cancer Patients: Results from the Ovarian Cancer Follow-Up Study, a Prospective Cohort Study
Source: Nutrients. 2023 Jan 31;15(3):717. doi: 10.3390/nu15030717 (PMC9920592; doi:10.3390/nu15030717)
Supplement: Supplementary file 1 [file nutrients-15-00717-s001.zip › nutrients-2107345-supplementary.pdf]

Supplementary Table S1. The components of NR, LIM, and NRF index scores *

Score	NR index score			LIM index score
	Macronutrients	Vitamins	Minerals	
**NRF6.3 index score**	Protein, fiber	A, C	Ca, Fe	SFA, Na, added sugar
**NRF9.3 index score**	Protein, fiber	A, C, E	Ca, Fe, Mg, K	SFA, Na, added sugar
**NRF11.3 index score**	Protein, fiber	A, B_{12}, C, E	Ca, Fe, Mg, Zn, K	SFA, Na, added sugar
**NRF15.3 index score**	MUFA, protein, fiber	A, B_{12}, C, D, E, thiamin, riboflavin, folate	Ca, Fe, Zn, K	SFA, Na, added sugar

Abbreviations: Ca, calcium; Fe, iron; K, potassium; LIM, limited nutrients; Mg, magnesium; MUFA, monounsaturated fatty acid; Na, sodium; NR, nutrients-rich; NRF, nutrients-rich food; SFA, saturated fatty acid; Zn, zinc.

* NR index scores are calculated through the sum of the content of NR in 100-kcal edible portion divided by the reference daily values for NR based on the 2000-kcal/d diet; LIM index score is calculated through the sum of content of LIM in 100-kcal edible portion divided by the reference daily values for LIM based on the 2000-kcal/d diet; NRF index scores are calculated through NR index scores subtracting LIM index score.

Supplementary Table S2. Selected clinical and immunohistochemical characteristics and associations with overall survival among ovarian cancer patients

Characteristics	No. of deaths/total (%)	Adjusted HR (95% CI)
**Clinical characteristics** *		
Age at diagnosis (year)		
≤ 50	45/258 (17.44)	1.00 (Ref)
> 50	85/445 (19.10)	1.24 (0.85-1.79)
Histological type		
Serous	92/479 (19.21)	1.00 (Ref)
Non-serous	38/224 (16.96)	1.71 (1.11-2.66)
Histopathologic grade		
Well differentiated	5/56 (8.93)	1.00 (Ref)
Moderately differentiated	7/48 (14.58)	1.12 (0.35-3.57)
Poorly differentiated	118/599 (19.70)	1.76 (0.70-4.43)
FIGO stage		
I-II	41/342 (11.99)	1.00 (Ref)
III-IV	89/338 (26.33)	2.54 (1.65-3.91)
Residual lesions		
No	82/553 (14.83)	1.00 (Ref)
< 1 cm	31/106 (29.25)	1.73 (1.11-2.68)

≥ 1 cm	17/44 (38.64)	2.41 (1.39-4.16)
Comorbidities		
No	74/393 (18.83)	1.00 (Ref)
Yes	56/310 (18.06)	0.97 (0.68-1.38)
**Immunohistochemical characteristics** †		
WT-1		
Positive	59/378 (15.61)	1.00 (Ref)
Negative	44/190 (23.16)	2.42 (1.51-3.87)
ER		
Positive	81/454 (17.84)	1.00 (Ref)
Negative	30/129 (23.26)	2.09 (1.24-3.52)
PR		
Positive	50/321 (15.58)	1.00 (Ref)
Negative	61/262 (23.28)	1.60 (1.07-2.38)
Vimentin		
Positive	29/156 (18.59)	1.00 (Ref)
Negative	65/359 (18.11)	0.84 (0.51-1.38)
p53		
Positive	92/473 (19.45)	1.00 (Ref)
Negative	27/151 (17.88)	0.94 (0.61-1.44)

Abbreviations: CI, confidence interval; ER, Estrogen Receptor; FIGO, International Federation of Gynecology and Obstetrics; HR, hazard ratio; PR,

Progestogen Receptor; Ref, reference; WT-1, Wilms' tumor-1.

[*] Mutually adjusted for all other clinical characteristics listed in the table.

[†] Adjusted for age at diagnosis (< 50 or ≥ 50 years), FIGO stage (I–II, III–IV, and unknown), histological type (serous or non-serous), histopathologic grade (well, moderately, and poorly differentiated), residual lesions (none, < 1, and ≥ 1 cm), and comorbidities (yes or no).

Supplementary Table S3. Data collection on the consumptions of vegetable and fruit

**Variables**	**Frequencies of consumption**						
**Vegetable**							
Chinese cabbage	≥ 2 times/day	1 times/day	4-6 times/week	2-3 times/week	1 times/week	2-3 times/month	almost never
Pakchoi	≥ 2 times/day	1 times/day	4-6 times/week	2-3 times/week	1 times/week	2-3 times/month	almost never
Kohlrabi	≥ 2 times/day	1 times/day	4-6 times/week	2-3 times/week	1 times/week	2-3 times/month	almost never
Rape	≥ 2 times/day	1 times/day	4-6 times/week	2-3 times/week	1 times/week	2-3 times/month	almost never
Spinach	≥ 2 times/day	1 times/day	4-6 times/week	2-3 times/week	1 times/week	2-3 times/month	almost never
Tomato	≥ 2 times/day	1 times/day	4-6 times/week	2-3 times/week	1 times/week	2-3 times/month	almost never
Pumpkin	≥ 2 times/day	1 times/day	4-6 times/week	2-3 times/week	1 times/week	2-3 times/month	almost never
Cucumber	≥ 2 times/day	1 times/day	4-6 times/week	2-3 times/week	1 times/week	2-3 times/month	almost never
Wax gourd	≥ 2 times/day	1 times/day	4-6 times/week	2-3 times/week	1 times/week	2-3 times/month	almost never
Celery	≥ 2 times/day	1 times/day	4-6 times/week	2-3 times/week	1 times/week	2-3 times/month	almost never
Eggplant	≥ 2 times/day	1 times/day	4-6 times/week	2-3 times/week	1 times/week	2-3 times/month	almost never
Broccoli	≥ 2 times/day	1 times/day	4-6 times/week	2-3 times/week	1 times/week	2-3 times/month	almost never
Cauliflower	≥ 2 times/day	1 times/day	4-6 times/week	2-3 times/week	1 times/week	2-3 times/month	almost never
Carrot	≥ 2 times/day	1 times/day	4-6 times/week	2-3 times/week	1 times/week	2-3 times/month	almost never

Other radishes	≥ 2 times/day	1 times/day	4-6 times/week	2-3 times/week	1 times/week	2-3 times/month	almost never
Pimento	≥ 2 times/day	1 times/day	4-6 times/week	2-3 times/week	1 times/week	2-3 times/month	almost never
Pepper	≥ 2 times/day	1 times/day	4-6 times/week	2-3 times/week	1 times/week	2-3 times/month	almost never
Agaric	≥ 2 times/day	1 times/day	4-6 times/week	2-3 times/week	1 times/week	2-3 times/month	almost never
Mushroom	≥ 2 times/day	1 times/day	4-6 times/week	2-3 times/week	1 times/week	2-3 times/month	almost never
Leeks	≥ 2 times/day	1 times/day	4-6 times/week	2-3 times/week	1 times/week	2-3 times/month	almost never
Onion	≥ 2 times/day	1 times/day	4-6 times/week	2-3 times/week	1 times/week	2-3 times/month	almost never
Garlic sprout	≥ 2 times/day	1 times/day	4-6 times/week	2-3 times/week	1 times/week	2-3 times/month	almost never
Seaweed	≥ 2 times/day	1 times/day	4-6 times/week	2-3 times/week	1 times/week	2-3 times/month	almost never
Lotus root	≥ 2 times/day	1 times/day	4-6 times/week	2-3 times/week	1 times/week	2-3 times/month	almost never
Jujube	≥ 2 times/day	1 times/day	4-6 times/week	2-3 times/week	1 times/week	2-3 times/month	almost never
Scallion	≥ 2 times/day	1 times/day	4-6 times/week	2-3 times/week	1 times/week	2-3 times/month	almost never
Garlic	≥ 2 times/day	1 times/day	4-6 times/week	2-3 times/week	1 times/week	2-3 times/month	almost never
Ginger	≥ 2 times/day	1 times/day	4-6 times/week	2-3 times/week	1 times/week	2-3 times/month	almost never
**Fruit**							
Apple	≥ 2 times/day	1 times/day	4-6 times/week	2-3 times/week	1 times/week	2-3 times/month	almost never

Pear	≥ 2 times/day	1 times/day	4-6 times/week	2-3 times/week	1 times/week	2-3 times/month	almost never
Orange	≥ 2 times/day	1 times/day	4-6 times/week	2-3 times/week	1 times/week	2-3 times/month	almost never
Banana	≥ 2 times/day	1 times/day	4-6 times/week	2-3 times/week	1 times/week	2-3 times/month	almost never
Watermelon	≥ 2 times/day	1 times/day	4-6 times/week	2-3 times/week	1 times/week	2-3 times/month	almost never
Peach	≥ 2 times/day	1 times/day	4-6 times/week	2-3 times/week	1 times/week	2-3 times/month	almost never
Pineapple	≥ 2 times/day	1 times/day	4-6 times/week	2-3 times/week	1 times/week	2-3 times/month	almost never
Kiwi fruit	≥ 2 times/day	1 times/day	4-6 times/week	2-3 times/week	1 times/week	2-3 times/month	almost never
Grape	≥ 2 times/day	1 times/day	4-6 times/week	2-3 times/week	1 times/week	2-3 times/month	almost never
Strawberry	≥ 2 times/day	1 times/day	4-6 times/week	2-3 times/week	1 times/week	2-3 times/month	almost never
Other fruits	≥ 2 times/day	1 times/day	4-6 times/week	2-3 times/week	1 times/week	2-3 times/month	almost never

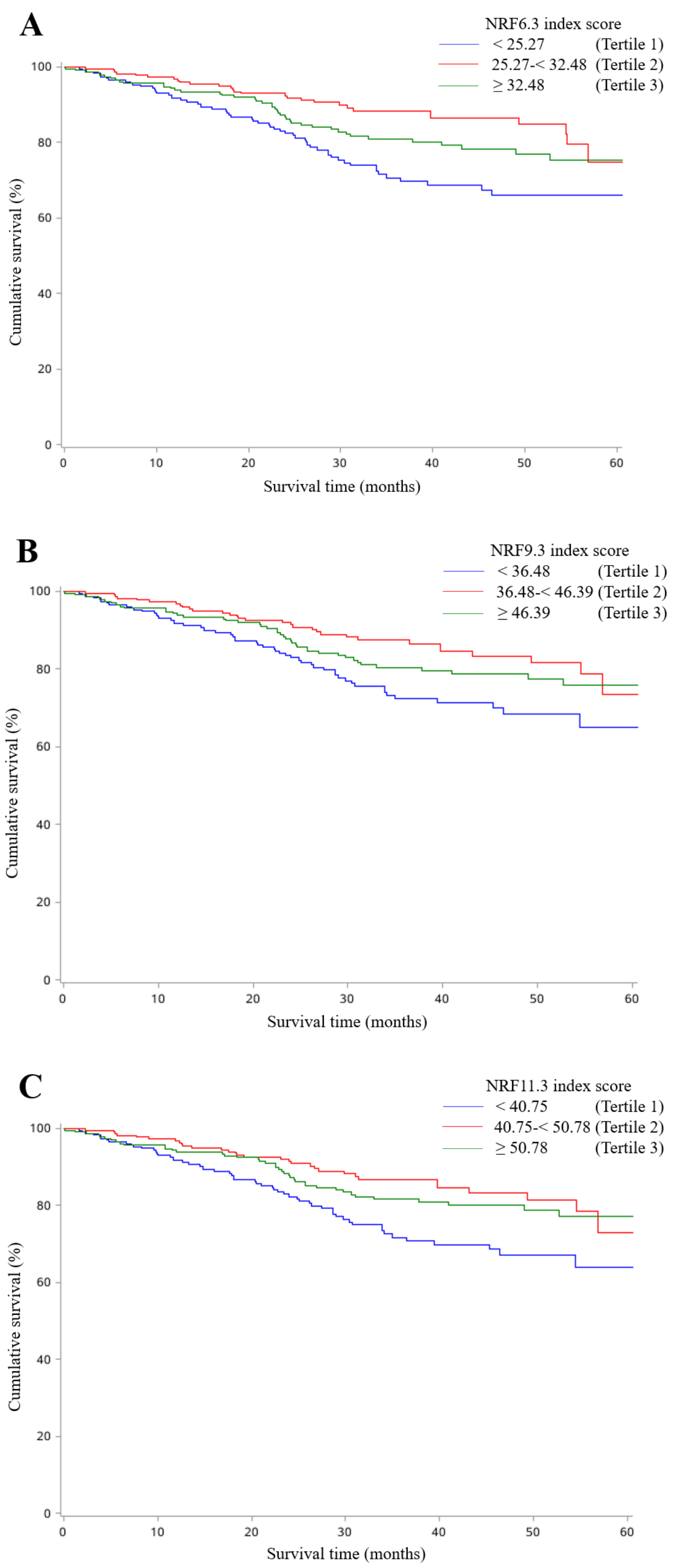

Supplementary Figure S1. Kaplan–Meier survival curves for NRF6.3 index score (A), NRF9.3 index score (B), NRF11.3 index score (C)

A

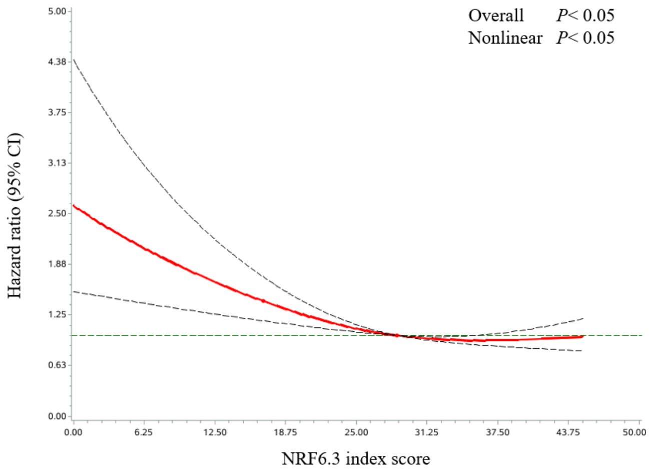

Overall P< 0.05
Nonlinear P< 0.05
Hazard ratio (95% CI)
5.00
4.38
3.75
3.13
2.50
1.88
1.25
0.63
0.00
0.00
6.25
12.50
18.75
25.00
31.25
37.50
43.75
50.00
NRF6.3 index score

B

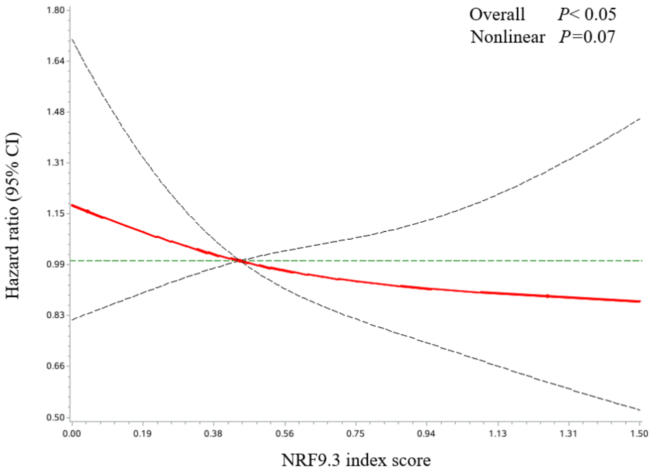

Overall P< 0.05
Nonlinear P=0.07
Hazard ratio (95% CI)
1.80
1.64
1.48
1.31
1.15
0.99
0.83
0.66
0.50
0.00
0.19
0.38
0.56
0.75
0.94
1.13
1.31
1.50
NRF9.3 index score

C

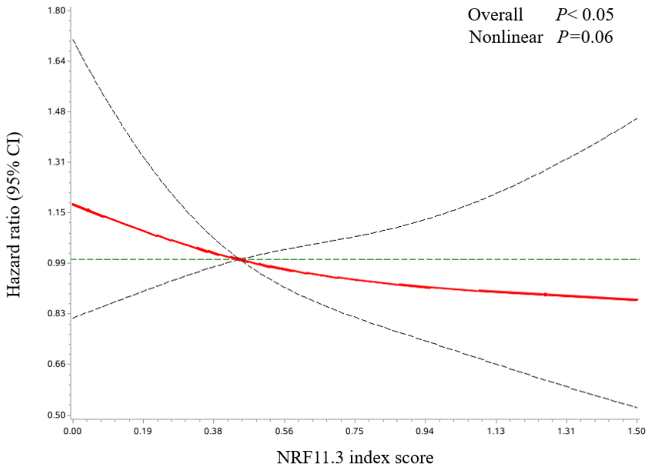

Overall P< 0.05
Nonlinear P=0.06
Hazard ratio (95% CI)
1.80
1.64
1.48
1.31
1.15
0.99
0.83
0.66
0.50
0.00
0.19
0.38
0.56
0.75
0.94
1.13
1.31
1.50
NRF11.3 index score

Supplementary Figure S2. HRs and 95% CIs of overall survival among OC patients by NRF6.3 index score (A), NRF9.3 index score (B), NRF11.3 index score (C). The associations were adjusted for age at diagnosis, education, cigarette smoking, alcohol drinking, monthly household income, dietary change, menopausal status, parity, body mass index, physical activity, FIGO stage, histological type, histopathologic grade, residual lesions, comorbidities, and total energy intake. The red line and dashed line represent the estimated HRs and their 95% CIs, respectively. Abbreviations: CI, confidence interval; HR, hazard ratio; OC, ovarian cancer.
